# Molecular epidemiology of canine GM1 gangliosidosis in the Shiba Inu breed in Japan: relationship between regional prevalence and carrier frequency

**DOI:** 10.1186/1746-6148-9-132

**Published:** 2013-07-03

**Authors:** Mohammad M Uddin, Sayaka Arata, Yukari Takeuchi, Hye-Sook Chang, Keijiro Mizukami, Akira Yabuki, Mohammad M Rahman, Moeko Kohyama, Mohammad A Hossain, Kenji Takayama, Osamu Yamato

**Affiliations:** 1 Laboratory of Clinical Pathology, Department of Veterinary Medicine, Joint Faculty of Veterinary Medicine, Kagoshima University, 1-21-24 Kohrimoto, Kagoshima, 890-0065, Japan; 2 Faculty of Veterinary Medicine, Chittagong Veterinary and Animal Sciences University, Chittagong, 4202, Bangladesh; 3 Laboratory of Veterinary Ethology, Department of Animal Resources Science, The University of Tokyo, 1-1-1 Yayoi, Bunkyo-ku, Tokyo, 113-8657, Japan; 4 Hayashiya Animal Medical Center, 18-6 Rokujizonara-cho, Uji-shi, Kyoto, 611-0001, Japan

**Keywords:** GM1 Gangliosidosis, Shiba Inu Dog, Molecular Epidemiology, Canine Inherited Disease

## Abstract

**Background:**

Canine GM1 gangliosidosis is a fatal disease in the Shiba Inu breed, which is one of the most popular traditional breeds in Japan and is maintained as a standard breed in many countries. Therefore, it is important to control and reduce the prevalence of GM1 gangliosidosis for maintaining the quality of this breed and to ensure supply of healthy dogs to prospective breeders and owners. This molecular epidemiological survey was performed to formulate an effective strategy for the control and prevention of this disease.

**Results:**

The survey was carried out among 590 clinically unaffected Shiba Inu dogs from the 8 districts of Japan, and a genotyping test was used to determine nation-wide and regional carrier frequencies. The number and native district of affected dogs identified in 16 years from 1997 to June 2013 were also surveyed retrospectively. Of the 590 dogs examined, 6 dogs (1.02%, 6/590) were carriers: 3 dogs (2.27%, 3/132) from the Kinki district and the other 3 dogs from the Hokkaido, Kanto, and Shikoku districts. The retrospective survey revealed 23 affected dogs, among which, 19 dogs (82.6%) were born within the last 7 years. Of the 23 affected dogs, 12 dogs (52.2%) were from the Kinki district. Pedigree analysis demonstrated that all the affected dogs and carriers with the pedigree information have a close blood relationship.

**Conclusions:**

Our results showed that the current carrier frequency for GM1 gangliosidosis is on the average 1.02% in Japan and rather high in the Kinki district, which may be related to the high prevalence observed over the past 16 years in this region. This observation suggests that carrier dogs are distributed all over Japan; however, kennels in the Kinki district may face an increased risk of GM1 gangliosidosis. Therefore, for effective control and prevention of this disease, it is necessary to examine as many breeding dogs as possible from all regions of Japan, especially from kennels located in areas with high prevalence and carrier frequency.

## Background

GM1 gangliosidosis, a lysosomal storage disease that affects the brain and multiple systemic organs, is caused by an autosomal recessively inherited deficiency in acid β-galactosidase, which is encoded by the *GLB1* gene [1]. GM1 gangliosidosis in Shiba Inu dogs was first reported in 2000 [2]. The causative mutation has been identified as a deletion of the cytosine in exon 15 at nucleotide position 1647 in the putative coding region (c.1647delC) of the canine *GLB1* gene [3], thereby enabling molecular diagnosis and/or genotyping with polymerase chain reaction (PCR)-based DNA tests [4,5]. Affected dogs manifest neurological signs of progressive motor dysfunction from 5–6 months of age and die at 12–18 months after a clearly defined clinical course [6,7], which is associated with progressive accumulation of GM1 ganglioside and the subsequent neuronal damage in the central nervous system [2,6,8].

The Shiba Inu breed is indigenous to and one of the most popular breeds in Japan, where it has been designated as a protected species since 1936 (http://www.nihonken-hozonkai.or.jp/). Thirty to forty thousand puppies are registered every year in Japan [9]. Shiba Inu dogs have been transported worldwide and are bred and maintained as a standard breed in many countries. Therefore, it is necessary to control and reduce the prevalence of GM1 gangliosidosis to maintain the quality of this traditional breed and to ensure supply of healthy dogs to prospective breeders and owners in Japan and other countries.

**Figure 1:**
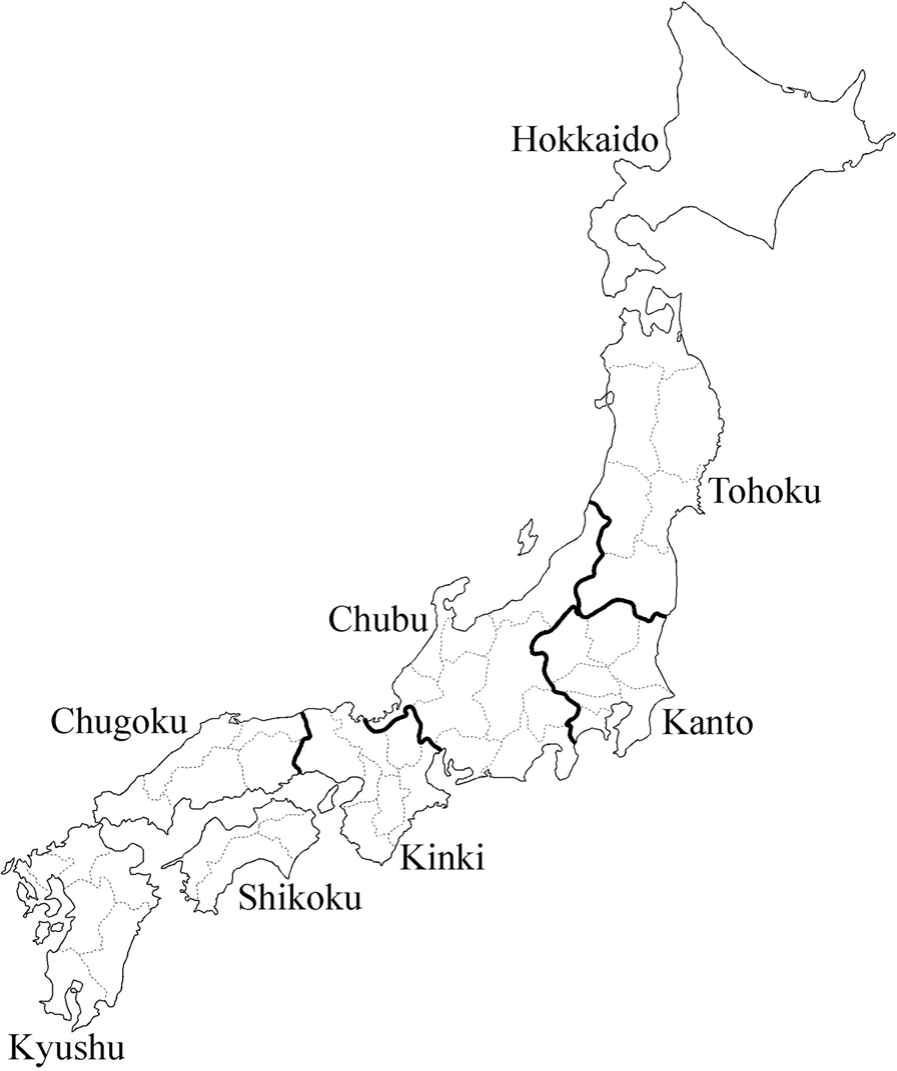
**Map of Japan showing the districts surveyed in the present study.** Bold lines show district boundaries on the main island, and dashed lines show prefectural boundaries.

Previously, a preliminary genotyping survey of the disease was carried out on 68 Shiba Inu dogs from only the Hokkaido and the Tohoku districts (Figure 1) in northern Japan [9]. This survey revealed 2 carriers among the 68 dogs. Although, carriers appear to be distributed all over Japan, specific areas and kennels may show high prevalence. Therefore, a large-scale and nationwide survey is necessary to know the current overall and regional prevalence and carrier frequency in Japan, as well as to formulate an effective strategy for the control and prevention of this disease.

In the present study, a large-scale molecular epidemiological survey of GM1 gangliosidosis in the Shiba Inu breed was carried out among 590 dogs from all the districts of Japan by using a genotyping test to determine the current overall and regional carrier frequency. The number and native district of affected dogs identified in 16 years from 1997 to June 2013 were also surveyed retrospectively. The pathway of transmission and distribution of the mutant allele were analyzed on the basis of pedigree information of the affected dogs and carriers identified previously and in this study. In this report, we also discuss the control and prevention of the disease on the basis of the results of these analyses.

## Results

### Carrier frequency

The results of the nationwide molecular epidemiological survey are shown in Table 1. This survey revealed 584 wild-type dogs, 6 carriers, and no affected dogs in the population of 590 animals. The carrier frequency in the population was 1.02%. Among the 6 carriers identified, 3 dogs were from the Kinki district and the other 3 dogs were from the Hokkaido, Kanto, and Shikoku districts.

**Table 1:** Results of the nationwide genotyping survey of GM1 gangliosidosis in Shiba Inu dogs in Japan

**District**	**Examined dogs**	**Genotypes**	
		**Wild-type**	**Carrier**
Hokkaido	23	22	1
Tohoku	17	17	0
Kanto	227	226	1
Chubu	135	135	0
Kinki	132	129	3
Chugoku	9	9	0
Shikoku	2	1	1
Kyushu*	42	42	0
Unknown	3	3	0
Total	590	584	6

* Including Okinawa prefecture, the small islands south of Kyushu Island.

### Retrospective survey and pedigree analysis

**Figure 2:**
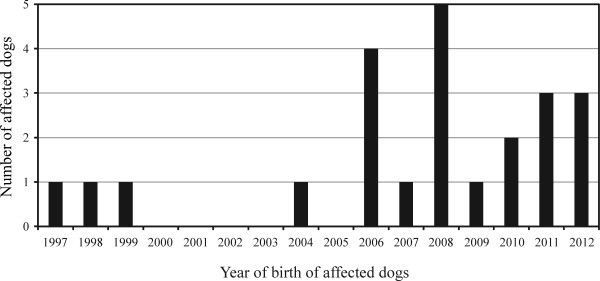
**Change in the number of Shiba Inu dogs with GM1 gangliosidosis from 1997 to 2012.** The 23 affected dogs (A1–A23) were plotted in the order in which they were born.

**Figure 3:**
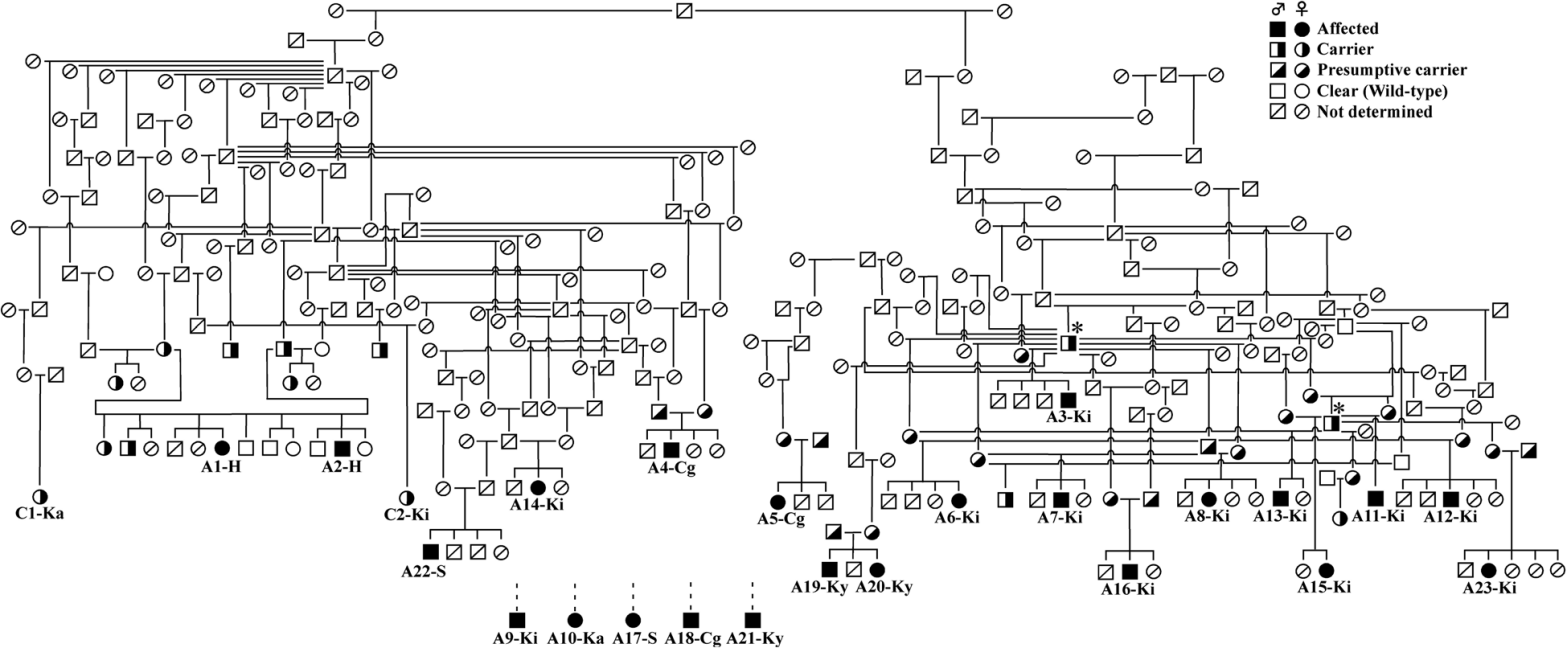
**The pedigree of Shiba Inu dogs with GM1 gangliosidosis.** The 23 affected dogs (A1–A23) are from the Hokkaido (H), Kinki (Ki), Chugoku (Cg), Kanto (Ka), Shikoku (S), and Kyushu (Ky) districts. Pedigree information was not available for 5 affected dogs (A9, A10, A17, A18, and A21). Two carriers (C1-Ka and C2-Ki) were identified in the Kanto and Kinki districts, respectively, in the molecular epidemiological survey of this study. * Carriers from a specific kennel, which are related to a number of affected and carrier offspring in the Kinki district.

The retrospective survey conducted using information collected over 16 years from 1997 to 2013 revealed 23 affected dogs (A1–A23) and several carriers that were related to these affected dogs. The first affected dog (A1-H), born in the Hokkaido district in 1997, was diagnosed with GM1 gangliosidosis histopathologically and biochemically [2], which was confirmed by molecular analysis using stored DNA and RNA [3]. Since this case, other affected dogs with the same molecular defect have been diagnosed sporadically until recently (Figure 2). Of the 23 affected dogs identified, 19 dogs were born within the last 7 years. The pedigree relationships among these affected dogs and the native district of each dog are shown in Figure 3. Although pedigree data were not available for 5 affected dogs, the remaining 18 affected dogs and several related carriers identified previously showed a blood relationship, according to the results of the pedigree analysis. Regarding the native districts, 12 affected dogs were from the Kinki region, accounting for approximately half of all the affected animals.

A pedigree analysis was also performed using the data from 2 carriers (C1-Ka and C2-Ki) identified in the present epidemiological survey. No pedigree data was available for the 4 remaining carriers. C1-Ka and C2-Ki also showed a blood relationship with the affected dogs and carriers identified in the retrospective survey (Figure 3).

## Discussion

Recessively inherited disorders such as lysosomal storage diseases are a major problem in the breeding of pure-breed domestic animals [9-11]. Identification of the carriers that have one abnormal allele in the gene pair, but that are normal in clinical appearance, is critical because there are no physical clues to the presence of the disease in these animals; however, the mutation is transmitted to half of their progeny [11]. The frequency of carriers in a population substantially exceeds the incidence of the disease; therefore, the presence of carriers makes recessive diseases the most difficult to control in pure-breed animals [11,12]. Canine GM1 gangliosidosis has been reported in other pure-breed dogs including English Springer Spaniels [13], Portuguese Water Dogs [14], and Alaskan Huskies [15]; however, to the best of our knowledge, a large-scale molecular epidemiological survey has not been performed to establish effective measures for the control and prevention of the disease.

Our results demonstrated that the overall carrier frequency of GM1 gangliosidosis among Shiba Inu dogs in Japan is 1.02% (6/590). In this study, regional data for northern (i.e., Hokkaido and Tohoku districts) and western or southern Japan (i.e., Chugoku, Shikoku, and Kyushu districts) were not fully evaluated because of the low number of samples collected from these regions. Among the districts sufficiently surveyed by a large number of samples, Kinki had the highest carrier frequency (2.27%, 3/132), which is more than 5 times that in Kanto (0.44%, 1/227). As the number of carriers is small, the calculated frequency could potentially be changed dramatically by chance. Specific families that include carriers may have also affected the frequency (i.e., population stratification). Therefore, the limitation of this study is that the calculated disease prevalence and carrier frequency may be influenced by chance.

In the retrospective survey, in the last 16 years, 23 affected dogs were identified, and 19 dogs of these were born within the last 7 years (Figure 2), suggesting a recent increase in the prevalence of the disease. Of the 23 affected dogs identified, 12 dogs were from the Kinki district. Of the 19 affected dogs identified in the last 7 years, 11 dogs were from the Kinki district, suggesting a recent increase in the prevalence in Japan, probably due to the high incidence of affected dogs in the Kinki district. The high incidence of affected dogs in the Kinki district seems to be attributable to a small number of popular carrier sires (shown by an asterisk in Figure 3) in the same region.

There are 8 district affiliates in Nihon-ken Hozon-kai, a major kennel club specializing in Japanese traditional breeds (http://www.nihonken-hozonkai.or.jp/). Furthermore, each district affiliate has several regional branch divisions with many kennels. Most kennels are small with only a few breeding dogs. Regional activities such as local dog-shows are carried out in each branch division before the district and nationwide activities. Therefore, there is a strong relationship among the kennels belonging to the same branch division. Mating also tends to be among dogs in the same branch division, resulting in potential inbreeding and homozygosity responsible for inherited disorders.

In the present study, a kennel with a very high prevalence of the disease was found in the Kinki district by pedigree analysis. This kennel housed 2 carriers, who were divisional champion dogs (shown by an asterisk in Figure 3), which produced many of the affected and carrier offspring in the Kinki district. Such kennels may be producing affected dogs sporadically by unknowingly mating carriers. It is thought that affected dogs are produced in a small proportion of kennels with high prevalence, and the mutant allele is then distributed to the surrounding areas from those kennels. This may be the reason behind the relationship between regional prevalence and carrier frequency in GM1 gangliosidosis in Shiba Inu dogs. A similar trend was observed in a survey of neuronal ceroid lipofuscinosis in the Border Collie breed in Japan [16]. Therefore, for the effective control and prevention of these fatal inherited diseases, it is necessary to examine as many breeding dogs as possible, especially from kennels located in areas with high prevalence and carrier frequency. In addition, genotyping of specimens from a random population of breeding dogs should be continued to detect sporadic carriers and prevent their use from being used as breeding dogs [12]. These approaches will also gradually decrease the number of dogs carrying the mutant allele. These active and continuous preventive measures may be necessary to eliminate GM1 gangliosidosis in Shiba Inu dogs.

## Conclusions

Based on the results of the present study, the current carrier frequency for GM1 gangliosidosis in the overall Shiba Inu dog population in Japan is 1.02%, and it is rather high (2.27%) in the Kinki district. The high carrier frequency in the Kinki region seems to be related to the high prevalence in this district. Therefore, for the effective control and prevention of this disease, it is necessary to examine as many breeding dogs as possible from all regions of Japan, especially from kennels located in areas with high prevalence and carrier frequency.

## Methods

### Sample collection and genotyping

Blood samples were collected randomly from 590 client-owned Shiba Inu-breed dogs with the cooperation of their owners and veterinarians from different animal hospitals in Japan. These dogs were clinically unaffected for GM1 gangliosidosis. The dogs were native to the Japanese districts shown in Table 1 and Figure 1. The genotypes of the dogs were determined using real-time PCR as previously reported [4].

### Retrospective survey and pedigree analysis

The number and the native district of the affected dogs were surveyed retrospectively based on the records in our laboratory, which has been exclusively supporting the diagnosis of this disease in Japan. Pedigree analysis was performed to elucidate the genetic relationships among the affected dogs and carriers identified previously and in the present study, as well as to deduce the pathway of transmission and distribution of the mutant allele. The genetic relationships among the affected dogs and carriers were analyzed using the pedigree records issued by the Nihon-ken Hozon-kai (http://www.nihonken-hozonkai.or.jp/) and the Japan Kennel Club (http://www.jkc.or.jp/), a kennel club certified by the Federation Cynologique Internationale (http://www.fci.be/).

All the experimental procedures using animals and their specimens were performed in accordance with the guidelines regulating animal use and ethics issued by the Experimental Animals Committee at Kagoshima University.

## Competing interests

The authors declare that they have no competing interests.

## Authors’ contributions

MMU analyzed the results and drafted the manuscript. SA and YT collected the blood from the animals and prepared the samples. HSC, KM, AY, MMR, MK, and MAH carried out the genotyping tests and contributed equally to this study. KT surveyed the kennel with high prevalence of the disease in the Kinki district. OY led the project, designed the study, and revised the results and manuscript. All authors read and approved the final manuscript.
